# The Impact of Plant-Based Diets on Dietary Acid Load Metrics in Venezuela: A Cross-Sectional Study

**DOI:** 10.3390/nu15122745

**Published:** 2023-06-14

**Authors:** Jesús Enrique Ekmeiro-Salvador, Maximilian Andreas Storz

**Affiliations:** 1 Postgraduate Department, Food Science, University of Oriente, Anzoátegui 6001, Venezuela; jekmeiro@gmail.com; 2 Department of Internal Medicine II, Centre for Complementary Medicine, Freiburg University Hospital Faculty of Medicine, University of Freiburg, 79106 Freiburg, Germany

**Keywords:** dietary acid load, potential renal acid load, plant-based diet, vegan, vegetarian, acid–base balance

## Abstract

Dietary acid load (DAL) is an important determinant of the acid–base balance in humans and has been associated with several chronic non-communicable diseases. Plant-based diets, including vegetarian and vegan diets, decrease DAL—although their alkalizing potential varies substantially. Their net effect on common DAL scores, including potential renal acid load and net endogenous acid production, has been insufficiently quantified and is poorly understood—particularly in populations outside of Europe and North America. We assessed the associations between three plant-based dietary patterns (flexitarian vs. lacto-ovo-vegetarian vs. vegan diet) and DAL scores in a healthy Venezuelan population in the metropolitan area of Puerto La Cruz, Venezuela. Substantial differences in DAL scores were observed, whereby the vegan diet yielded the highest alkalizing potential, followed by the lacto-ovo-vegetarian and the flexitarian diet. DAL scores were substantially lower in comparison to European and North American plant-based populations, probably due to the higher potassium intake (exceeding 4000 mg/d in vegans), the higher magnesium intake (390.31 ± 1.79 mg/d in vegans) and the lower intake of protein in vegans and lacto-ovo-vegetarians. Additional studies in other non-industrialized populations are warranted to allow for a better understanding of the (numeric) impact of plant-based dietary patterns on DAL scores, potentially allowing for an establishment of reference ranges in the near future.

## 1. Introduction

Diet composition may affect acid–base balance in humans by providing acid or base precursors [1,2,3]. Diets abundant in alkalizing plant foods may decrease the dietary acid load (DAL), whereas acidifying foods including eggs, meat and cheese may increase it [1,4]. Contemporary Western diets lack sufficient amounts of plant-based foods, which contain important base precursors such as potassium alkali salts and magnesium [1,5]. Instead, they include supraphysiological amounts of acid precursors, mainly in the form of sulfur containing amino acids and phosphate additives [6,7].

This imbalance in acid and base precursors may translate into a high DAL, which has been associated with chronic low-grade inflammation and tissue damage [8,9]. The typical acid load of Western diets ranges from 60 to 100 mEq per day and has been associated with numerous adverse chronic health conditions [10,11], including cardiovascular disease, chronic kidney disease, type 2 diabetes and certain cancers [12,13,14,15,16]. A high DAL may further contribute to sarcopenia and frailty [17,18,19], reduced bone mineral density [19,20,21] and hypertension [22,23].

Dietary modifications are an effective means to reduce DAL [5]. Plant-based diets (PBDs) in particular may decrease DAL [24], whereas carbohydrate restriction may have unfavorable effects [25]. One metric to estimate DAL is the PRAL (potential renal acid load) score [26], an estimation formula that considers intestinal absorption rates for protein and several micronutrients including magnesium, potassium, calcium and phosphorus. Positive PRAL scores (>0 mEq/d) suggest an acidifying diet, whereas negative PRAL scores (<0 mEq/d) suggest an alkalizing diet [5].

Lacto-ovo-vegetarian (LOV) and vegan diets often yield negative PRAL scores, indicating alkalizing effects to a various degree [1,5]. Notably, some studies suggest that both diets differ substantially with regard to their alkalizing potential [5,7]. LOV diets generally tend to be less alkaline than vegan diets—depending on the particular diet composition. A reduction in the consumption of industrialized products with phosphorus additives (carbonated beverages, industrialized teas, meats, frozen products and other products with food additives) generally reduces DAL [19].

It is noteworthy that DAL has been rarely assessed in lacto-ovo-vegetarians and vegans outside of highly industrialized nations. The majority of studies were conducted in the United States of America or Europe, while studies from other countries in which plant-based nutrition is practiced are urgently warranted to gain a better and more quantitative understanding of the acid–base impact of said diets [5].

Addressing this gap in the literature, we performed a post hoc analysis of a Venezuelan cross-sectional study and investigated DAL scores in a well-characterized sample of *n* = 224 individuals reporting adherence to a PBD [27]. Venezuela is a particularly salient case, because the country is currently experiencing a socioeconomic crisis with potential implications for food security and food shortages [28].

The main purpose of our study was to compare three different plant-based dietary groups with regard to their DAL impact: flexitarians, lacto-ovo-vegetarians and vegans.

Based on previous work in the field, we hypothesized that vegans would yield the lowest DAL scores, followed by lacto-ovo-vegetarians and flexitarians. Finally, we sought to contrast our results to DAL scores found in plant-based European and Northern American populations.

## 2. Materials and Methods

### 2.1. Study Background 

The study methods for the primary data acquisition of this cross-sectional study have been described elsewhere in great detail [27]. In brief, we recruited *n* = 224 individuals that reported the consumption of a PBD between July 2018 and February 2020 in the metropolitan area of Puerto La Cruz in Venezuela, South America. The sample was further divided into vegans, lacto-ovo-vegetarians and flexitarians based on food intake reported in two independent 24 h dietary recalls and based on the following definitions in Table 1.

The two dietary recalls were collected on non-consecutive days of the same week for each participant. All information was collected during face-to-face interviews. Interviews were conducted by previously trained nutritionists (also called “nutricionistas dietistas”) using an established multi-step approach [27]. The employed questionnaires in this study were standardized and specific characteristics were reported previously [27]. The involved study team transformed the reported food measurements and portions into grams and milliliters using Venezuelan measurement tables and portions (UCV School of Nutrition and Dietetics, 2002). Food Processor^®^ software (version 10) was then subsequently used to calculate macro- and micronutrient intake from those foods (based on the current Venezuelan Food Composition Table (Instituto Nacional de Nutrición [INN], 2015) [29]. 

The major initial aim of the study was to learn more about the plant-based community in Venezuela, including its motivations for adopting plant-based nutrition as well as adherence to plant-based dietary patterns and their nutritional adequacy. A specific power analysis for the present secondary data analysis was not performed. The study received approval from the local Bioethics and Research Committee [27]. All participants gave written and oral consent to participate.

### 2.2. Dietary Acid Load Estimations 

DAL was estimated using established and widely used concepts, including PRAL and NEAP (Net Endogenous Acid Production) [26]. These employed scores included the PRAL formula by Remer et al. (hereafter called PRAL_R_) [26], the NEAP formula by Remer et al. (hereafter called NEAP_R_) [30] and the NEAP formula proposed by Frassetto et al. (hereafter called NEAP_F_) [31].

PRAL_R_ was estimated as follows [26]:

PRAL_R_ (mEq/day) = (0.49 × total protein (g/day)) + (0.037 × phosphorus (mg/day)) − (0.021 × potassium (mg/day)) − (0.026 × magnesium (mg/day)) − (0.013 × calcium (mg/day))

NEAP_R_ was estimated as follows [30]:

Estimated NEAP_R_ (mEq/d) = PRAL (mEq/d) + OAest (mEq/d)

OAest was calculated as follows:

Individual body surface area × 41/1.73

NEAP_F_ was estimated as follows [31]:

NEAP_F_ (mEq/d) = (54.4 × protein (g/d)/potassium (mEq/d)) − 10.2

The PRAL_R_ formula considers ionic dissociation and sulfur metabolism. The formula was previously validated against urinary renal net acid excretion and shown to reliably estimate the acid load from diet composition [1]. All three formulas are commonly used in epidemiological and clinical research with varying but adequate performance and accuracy, as recently reported by Parmenter et al. [32,33].

### 2.3. Statistical Analysis 

We used Stata 14 statistical software (StataCorp. 2015. Stata Statistical Software: Release 14. College Station, TX, USA: StataCorp LP) for our statistical analysis. Histograms and box plots were used to check for the normality of the data. Moreover, we made use of Stata’s Shapiro–Francia Test to test for normality. The variables of interest were all normally distributed and thus described with their mean and standard deviation. Analysis of variance was subsequently used to test for statistically significant differences in the means of nutrients and DAL scores between the 3 dietary groups. This was followed by Stata’s pairwise comparisons command in order to detect which groups statistically differed from each other. Groups that differed statistically significantly from each other were explicitly marked using superscript symbols. Finally, multiple regression models were run to predict the PRAL_R_, NEAP_R_ and NEAP_F_, respectively, from the dietary category, sex and body mass index (BMI). All tests were two-tailed, and a *p*-value < 0.05 was used as a cutoff for statistical significance. Finally, we used scatterplots and contour plots to visualize the relationship between dietary intake and selected DAL scores.

## 3. Results

**Figure 1:**
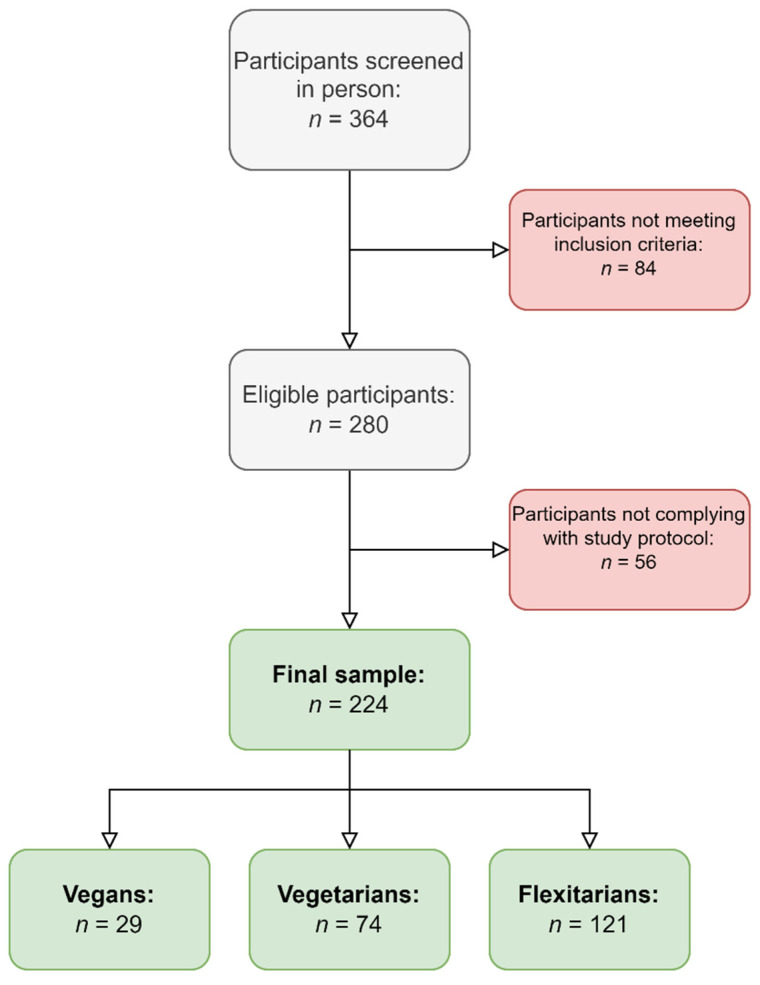
Participant inclusion flowchart.

The study sample comprised *n* = 29 vegans, *n* = 74 lacto-ovo-vegetarians and *n* = 121 flexitarians. Figure 1 shows a participant inclusion flowchart.

Approximately 38% of the study population were male (*n* = 84), and about 62% were female (*n* = 140). The mean age in the flexitarian group was 40.20 ± 11.54 years, 36.19 ± 9.13 years in the vegetarian group and 33.80 ± 10.59 years in the vegan group. Additional sociodemographic information describing the examined sample may be obtained from Ekemiro-Salvador et al. [27].

Table 2 displays the nutrient intake in this particular sample. Flexitarians consumed significantly higher amounts of protein than vegetarians and vegans (*p* < 0.001). Their intake amount was almost twice as high compared to vegans. A same trend was observed for total energy intake and fat intake (*p* < 0.001). In contrast, vegans yielded the highest potassium intake and had the lowest phosphorus intake (*p* < 0.001).

Table 3 displays differences in the three examined DAL scores between the three dietary groups. We observed statistically significant intergroup differences with regard to all three DAL metrics. Vegans generally yielded the lowest DAL scores. The PRAL value of −42.65 ± 11.35 in vegans deserves special consideration, as it indicates a strong alkalizing potential. NEAP scores in flexitarians were roughly twice as high as in lacto-ovo-vegetarians. The same applied for PRAL_R_ and NEAP_F_ when comparing lacto-ovo-vegetarians to vegans.

**Figure 2:**
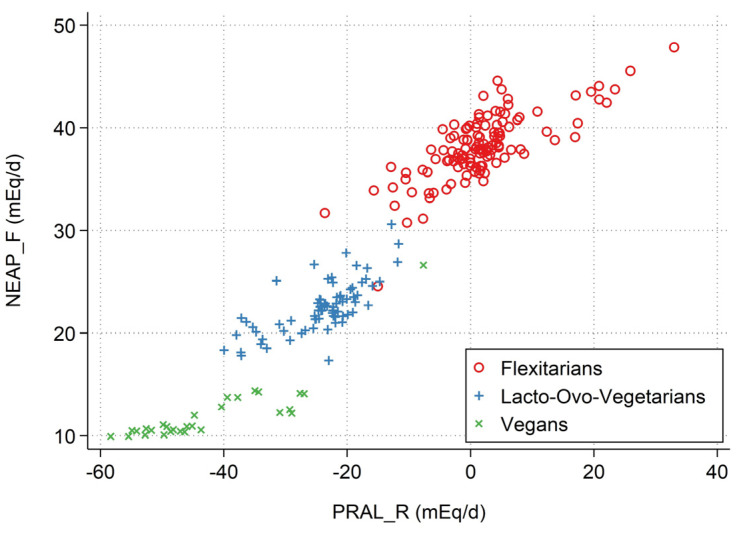
Scatter plot displaying the relationship between both nutrient-intake based DAL estimates (PRAL_R_ on the x-axis vs. NEAP_F_ on the y-axis).

A Pearson’s product-moment correlation was run to assess the relationship between PRAL_R_ and NEAP_F_ in the entire sample. As expected, there was a strong positive correlation between both DAL estimates; r = 0.95, *p* < 0.0001. Figure 2 visually displays this strong correlation between both nutrient-based DAL estimates (PRAL_R_ vs. NEAP_F_) and highlights the large DAL differences in the sample.

**Figure 3:**
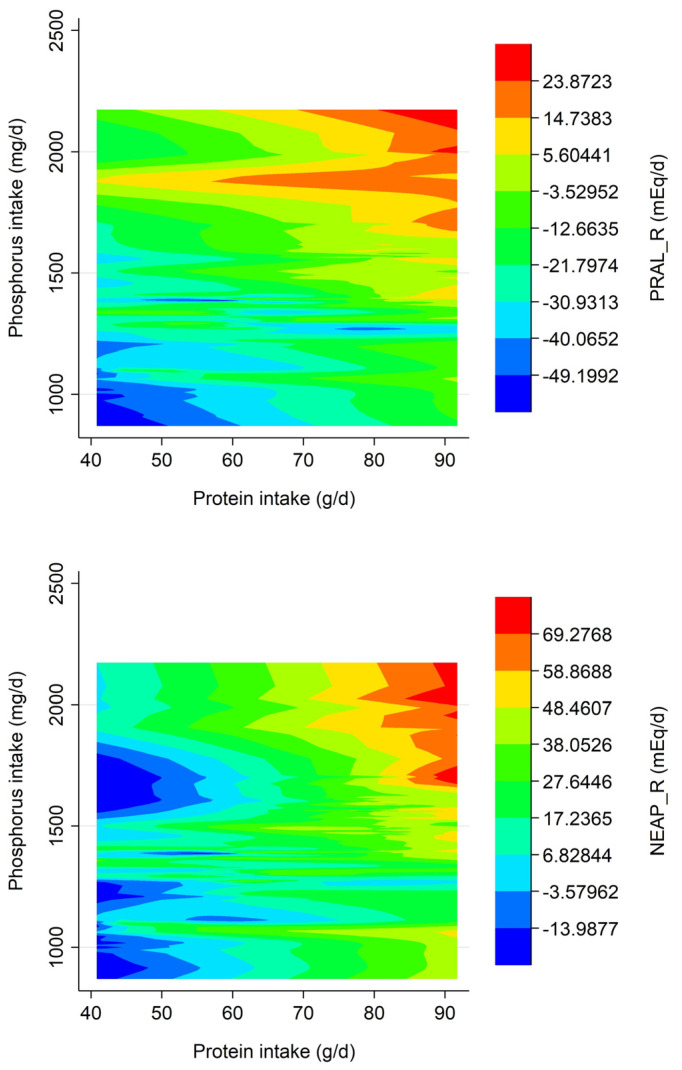
Contour plots displaying the relationship between protein intake (x-axis), phosphorus intake (y-axis) and the two DAL scores by Remer et al. (z-axis).

Figure 3 shows two contour plots to visualize the relationship between protein intake, phosphorus intake (which both contribute acid precursors) and the two DAL estimates by Remer et al. (PRAL_R_ and NEAP_R_) [26]. PRAL_R_ is shown at the top, whereas NEAP_R_ is shown at the bottom. Both plots highlight the importance of protein as a main DAL contributor.

Table 4 shows the employed multivariate linear regression models examining potential associations between PRAL_R_, NEAP_R_, NEAP_F_ and diet category. Crude associations are shown in model 1, whereas model 2 shows associations after adjustments for covariates (sex and BMI). Vegans and lacto-ovo-vegetarians yielded significantly lower DAL scores as compared to flexitarians, and this association remained significant even after adjustment for important covariates.

## 4. Discussion

DAL is an emerging diet quality marker of current epidemiological and clinical interest [5]. A high DAL has been associated with various health repercussions and adverse clinical outcomes, including hyperuricemia [34], an unfavorable lipid profile [35] and all-cause mortality [36]. As such, some medical practitioners and dieticians aim to reduce the acid load burden from diet with targeted dietary interventions. While some practitioners prefer specific neutraceuticals or base-precursor-enriched supplements, others aim towards larger dietary modifications. This may be particularly the case in the field of nephrology, where patients with chronic kidney disease are supposed to benefit from a more alkaline diet [37,38]. 

Plant-based diets are an effective and proven means to reduce DAL [7], yet their numeric impact on DAL metrics is insufficiently quantified and poorly understood—particularly in plant-based populations outside of North America and Europe [5].

The majority of studies investigating DAL in vegan and vegetarian individuals were conducted in highly industrialized nations, in particular in the United States of America [1,39,40], Germany [41,42] and Belgium [43]. Studies in non-Western and non-industrialized populations are, as of yet, scarce. To the best of our knowledge, we present the first analysis to examine DAL scores in Venezuelan and Latin-American plant-based consumers. Comparing flexitarians, lacto-ovo-vegetarians and vegans, we found significant and substantial intergroup differences between the three examined dietary patterns.

As somewhat expected, vegans yielded substantially lower DAL scores than lacto-ovo-vegetarians in our study (*p* < 0.001). Hereby, the PRAL_R_ score of −42.65 ± 11.35 mEq/d in vegans suggests a strong alkalizing potential. The PRAL_R_ score of lacto-ovo-vegetarians also suggests an alkalizing potential (−24.24 ± 6.47 mEq/d), although less pronounced. The findings for vegans are essentially in line with results previously reported by other authors from Europe. Ströhle et al. reported a mean PRAL_R_ score of −39 ± 29 mEq/d in vegans from the German Vegan Study [41]. Compared to the remaining studies, however, the observed PRAL_R_ scores in our study were much lower.

Knurick et al. investigated DAL scores in US-based, non-obese, non-smoking vegans aged 19–50 years with at least 1 year of dietary adherence [40]. They reported a mean PRAL_R_ score of −15.2 ± 40.5 mEq/d in their cohort—a value that is closer to the mean PRAL_R_ score of lacto-ovo-vegetarians in our study. The same applied to a post hoc analysis by Müller et al., which showed a mean PRAL_R_ score of −23.57 ± 23.87 mEq/d in vegans after a 3-week dietary intervention [42].

One reason that could explain the very low DAL metrics in vegans in our cohort could be the combined effect of a relatively low protein intake (41.78 ± 3.39 g/d) and a high potassium intake exceeding the 4000 mg margin (4000.65 ± 255.03 mg/d) (Table 2). Protein in particular has the highest weighing factor in the PRAL_R_ formula by Remer et al. [26] and may account to a large extent for the observed intergroup differences. In this context, it is also worth mentioning that the number of vegans not meeting the Venezuelan protein and energy intake recommendations was substantially higher than in lacto-ovo-vegetarians and flexitarians (although not statistically significant).

The noticeable differences in DAL scores compared to North American and European studies may be interpreted as a reminder that it is necessary to have a close look at macro- and micronutrient intake to reliably judge the acidifying/alkalizing potential of a specific plant-based dietary pattern. Although vegan dietary patterns exclude animal protein, they may include other acidifying foods to various extents (such as processed grains or processed vegan junk food). Prominent examples include Quorn burgers or Quorn mince, which both have a PRAL value of approximately 9 mEq per 100 g edible portion [43]. The unavailability of said processed foods in Venezuela could also partly explain the difference in DAL scores across studies and the lower DAL scores in our cohort.

Our results allow for deeper insights into the alkalizing potential of plant-based dietary patterns and constitute an important step towards establishing reference values and to gain a better numeric understanding of PRAL_R_ scores in vegetarians and vegans. That said, a reservation must be made that some studies also suggested that a too-alkalizing diet may be potentially harmful [36,44]. As discussed in our previous review [5], excess diet alkalinity and acidity both showed weak associations with higher mortality in Swedish adults [44]. Comparable findings have been reported in an Iranian study using data from the Golestan Cohort Study [36].

Due to the cross-sectional nature of our study, we were unable to evaluate any associations with prospective health outcomes. This is a potential weakness that could be addressed with future prospective investigations.

Our analysis has several other strengths and weaknesses that warrant a further discussion. The strengths include the moderate sample size (*n* = 224) and the inclusion of three different DAL metrics (PRAL_R_, NEAP_R_ and NEAP_F_). As mentioned earlier, we are the first group to examine DAL scores in Venezuelan flexitarians, vegetarians and vegans. A major weakness is the lack of urinary acid load markers, as recently reported by Penczynski et al. [45]. For additional insights, researchers were recently encouraged to collect measures of urinary PRAL and NEAP; however, said measurements were not part of our study. In addition, we had no detailed information on the protein quality and amino acid distribution available for our sample. The latter would have allowed for additional insights into the diet composition of our sample.

Moreover, the cross-sectional nature of our study did not allow for causal interferences. The lack of a specific food group analysis is another major limitation that was not possible due to external factors. Finally, we acknowledge the low number of *n* = 29 vegans in our sample (when compared to the modest sample size in total). Then again, larger studies were hardly realizable in light of the current difficult sociopolitical context in Venezuela and the unavailability of funding for official research. Said contextual factors and the fact that the Venezuelan plant-based community is not as uniformly organized as, for example, in many European countries (where associations and societies for plant-based nutrition exist) constituted an important study recruitment barrier. In this regard, it is difficult to perform large-scale studies in Venezuelan vegans and vegetarians. Nevertheless, we believe our results to be important despite the aforementioned limitations.

## 5. Conclusions

Our results support the concept that plant-based diets are an effective means to reduce DAL scores. We found statistically significant and clinically relevant differences between the three examined dietary patterns, particularly when contrasting our results to Western vegan and vegetarian populations. Future studies in non-Western plant-based populations are therefore warranted to investigate (and confirm) the impact of said diets on acid–base balance, preferably supported by urinary and food group analyses. The latter is particularly warranted since the availability of plant products can be different depending on the geographical location and season, thus requiring local studies that offer the bases for local and more focused conclusions.

## Figures and Tables

**Table 1:** Definitions of the plant-based dietary patterns in this study.

Dietary Pattern	Definition
Vegan	No consumption of products of animal origin.
Lacto-ovo-vegetarian	No consumption of meat but consumption of other products of animal origin (eggs and dairy products) that do not involve animal sacrifice.
Flexitarian	Diets based mainly on products of plant origin but occasionally including some products of animal origin and possibly small amounts of meat, especially marine animals.

**Table 2:** Nutrient intake by plant-based dietary pattern: an overview.

Nutrient	Flexitarians (*n* = 121)	Lacto-Ovo-Vegetarians (*n* = 74)	Vegans (*n* = 29)	*p*-Value
Energy intake (kcal/d)	2189.07 ± 78.79	2091.39 ± 109.38	1881.14 ± 130.45 *^,^**^,^***	*p* < 0.001
Protein (g/d)	81.84 ± 4.04	59.68 ± 2.93	41.78 ± 3.39 *^,^**^,^***	*p* < 0.001
Carbohydrate (g/d)	296.04 ± 15.12	313.30 ± 22.45	301.23 ± 18.56 *^,^***	*p* < 0.001
Fat (g/day)	76.14 ± 5.91	67.03 ± 3.01	56.19 ± 6.21 *^,^**^,^***	*p* < 0.001
Magnesium (mg/d)	354.98 ± 1.23	377.71 ± 4.87	390.31 ± 1.79 *^,^**^,^***	*p* < 0.001
Calcium (mg/d)	876.20 ± 3.55	802.16 ± 10.24	756.89 ± 15.89 *^,^**^,^***	*p* < 0.001
Potassium (mg/d)	3598.34 ± 155.61	3890.75 ± 257.62	4000.65 ± 255.03 *^,^**^,^***	*p* < 0.001
Phosphorus (mg/d)	1563.41 ± 171.00	1310.11 ± 82.52	1104.8 ± 162.46 *^,^**^,^***	*p* < 0.001

* indicates statistically significant differences between flexitarians and lacto-ovo-vegetarians; ** indicates statistically significant differences between flexitarians and vegans; *** indicates statistically significant differences between lacto-ovo-vegetarians and vegans.

**Table 3:** DAL scores by plant-based dietary pattern: an overview.

DAL Metric	Flexitarians (*n* = 121)	Lacto-Ovo-Vegetarians (*n* = 74)	Vegans (*n* = 29)	*p*-Value
PRAL_R_ (mEq/d)	1.76 ± 8.36	−24.24 ± 6.47	−42.65 ± 11.35 *^,^**^,^***	*p* < 0.001
NEAP_R_ (mEq/d)	40.48 ± 11.45	14.24 ± 8.22	−6.77 ± 13.18 *^,^**^,^***	*p* < 0.001
NEAP_F_ (mEq/d)	38.15 ± 0.29	22.47 ± 0.30	12.11 ± 0.59 *^,^**^,^***	*p* < 0.001

* indicates statistically significant differences between flexitarians and lacto-ovo-vegetarians; ** indicates statistically significant differences between flexitarians and vegans; *** indicates statistically significant differences between lacto-ovo-vegetarians and vegans.

**Table 4:** Multivariate linear regression models examining potential associations between PRAL_R_, NEAP_R_, NEAP_F_ (dependent variable, top, middle and bottom) and diet category.

Independent Variables	β	SE	*p*	β	SE	*p*
	**PRAL_R_**					
	Model I			Model II		
Diet Category						
Flexitarian	-	-	-	-	-	-
Lacto-Ovo-Vegetarian	−26.00	1.22	<0.001	−25.75	1.10	<0.001
Vegan	−44.42	1.71	<0.001	−42.82	1.60	<0.001
Sex						
Female				−3.06	1.04	0.003
Male				-	-	-
Body mass index				0.89	0.14	<0.001
	**NEAP_R_**					
	Model I			Model II		
Diet Category						
Flexitarian	-	-	-	-	-	-
Lacto-Ovo-Vegetarian	−26.24	1.59	<0.001	−25.85	1.13	<0.001
Vegan	−47.25	2.22	<0.001	−42.79	1.65	<0.001
Sex						
Female				−8.89	1.07	<0.001
Male				-	-	-
Body mass index				1.58	0.15	<0.001
	**NEAP_F_**					
	Model I			Model II		
Diet Category						
Flexitarian	-	-	-	-	-	-
Lacto-Ovo-Vegetarian	−15.68	0.44	<0.001	−15.58	0.38	<0.001
Vegan	−26.04	0.62	<0.001	−24.99	0.56	<0.001
Sex						
Female				−1.51	0.36	<0.001
Male				-	-	-
Body mass index				0.36	0.05	<0.001

Model I shows crude associations. Model II is adjusted for sex and BMI. Significant regression equations were found for PRAL_R_ (F(4,219) = 291.45, *p* < 0.001), with an R2 of 0.84; for NEAP_R_ (F(4,219) = 336.91, *p* < 0.001), with an R2 of 0.86; and for NEAP_F_ (F(4,219) = 821.79, *p* < 0.001) with an R2 of 0.94.

## Data Availability

The datasets used and analyzed during the current study are available from the corresponding author upon reasonable request.

## References

[1] Kahleova H., McCann J., Alwarith J., Rembert E., Tura A., Holubkov R., Barnard N.D. (2021). A Plant-Based Diet in Overweight Adults in a 16-Week Randomized Clinical Trial: The Role of Dietary Acid Load. Clin. Nutr. ESPEN.

[2] Betz M.V., Nemec K.B., Zisman A.L. (2022). Plant-Based Diets in Kidney Disease: Nephrology Professionals’ Perspective. J. Ren. Nutr..

[3] Asplin J.R. (2022). Neglected Analytes in the 24-h Urine: Ammonium and Sulfate. Curr. Opin. Nephrol. Hypertens..

[4] Williams R.S., Kozan P., Samocha-Bonet D. (2016). The Role of Dietary Acid Load and Mild Metabolic Acidosis in Insulin Resistance in Humans. Biochimie.

[5] Storz M.A., Ronco A.L., Hannibal L. (2022). Observational and Clinical Evidence That Plant-Based Nutrition Reduces Dietary Acid Load. J. Nutr. Sci..

[6] Adeva M.M., Souto G. (2011). Diet-Induced Metabolic Acidosis. Clin. Nutr..

[7] Storz M.A., Ronco A.L. (2022). Reduced Dietary Acid Load in U.S. Vegetarian Adults: Results from the National Health and Nutrition Examination Survey. Food Sci. Nutr..

[8] Carnauba R.A., Baptistella A.B., Paschoal V., Hübscher G.H. (2017). Diet-Induced Low-Grade Metabolic Acidosis and Clinical Outcomes: A Review. Nutrients.

[9] Robey I.F. (2012). Examining the Relationship between Diet-Induced Acidosis and Cancer. Nutr. Metab..

[10] Gannon R.H.T., Millward D.J., Brown J.E., Macdonald H.M., Lovell D.P., Frassetto L.A., Remer T., Lanham-New S.A. (2008). Estimates of Daily Net Endogenous Acid Production in the Elderly UK Population: Analysis of the National Diet and Nutrition Survey (NDNS) of British Adults Aged 65 Years and Over. Br. J. Nutr..

[11] Lemann J. (1998). Relationship between Urinary Calcium and Net Acid Excretion as Determined by Dietary Protein and Potassium: A Review. Nephron.

[12] Ostrowska J., Janiszewska J., Szostak-Węgierek D. (2020). Dietary Acid Load and Cardiometabolic Risk Factors—A Narrative Review. Nutrients.

[13] Tariq A., Chen J., Yu B., Boerwinkle E., Coresh J., Grams M.E., Rebholz C.M. (2022). Metabolomics of Dietary Acid Load and Incident Chronic Kidney Disease. J. Ren. Nutr..

[14] Hatami E., Abbasi K., Salehi-sahlabadi A., Beigrezaei S., Bahrami A., Ghiasvand R., Pourmasoumi M. (2022). Dietary Acid Load and Risk of Type 2 Diabetes Mellitus: A Case–Control Study. Clin. Nutr. ESPEN.

[15] Ronco A.L., Martínez-López W., Calderón J.M., Storz M.A. (2022). Dietary acid load and esophageal cancer risk: A case-control study. Thorac. Cancer.

[16] Ronco A.L., Storz M.A., Martínez-López W., Calderón J.M., Golomar W. (2021). High dietary acid load is associated with prostate cancer risk: An epidemiological study. World Cancer Res. J..

[17] Chan R., Leung J., Woo J. (2015). Association Between Estimated Net Endogenous Acid Production and Subsequent Decline in Muscle Mass Over Four Years in Ambulatory Older Chinese People in Hong Kong: A Prospective Cohort Study. J. Gerontol. A Biol. Sci. Med. Sci..

[18] Welch A.A., MacGregor A.J., Skinner J., Spector T.D., Moayyeri A., Cassidy A. (2013). A Higher Alkaline Dietary Load Is Associated with Greater Indexes of Skeletal Muscle Mass in Women. Osteoporos. Int..

[19] Osuna-Padilla I.A., Leal-Escobar G., Garza-García C.A., Rodríguez-Castellanos F.E. (2019). Dietary Acid Load: Mechanisms and Evidence of Its Health Repercussions. Nefrología.

[20] Remer T., Manz F., Alexy U., Schoenau E., Wudy S.A., Shi L. (2011). Long-Term High Urinary Potential Renal Acid Load and Low Nitrogen Excretion Predict Reduced Diaphyseal Bone Mass and Bone Size in Children. J. Clin. Endocrinol. Metab..

[21] De Jonge E.A.L., Koromani F., Hofman A., Uitterlinden A.G., Franco O.H., Rivadeneira F., Kiefte-de Jong J.C. (2017). Dietary Acid Load, Trabecular Bone Integrity, and Mineral Density in an Ageing Population: The Rotterdam Study. Osteoporos. Int..

[22] Zhang L., Curhan G.C., Forman J.P. (2009). Diet-Dependent Net Acid Load and Risk of Incident Hypertension in United States Women. Hypertension.

[23] Krupp D., Esche J., Mensink G.B.M., Klenow S., Thamm M., Remer T. (2018). Dietary Acid Load and Potassium Intake Associate with Blood Pressure and Hypertension Prevalence in a Representative Sample of the German Adult Population. Nutrients.

[24] Galchenko A., Gapparova K., Sidorova E. (2023). The Influence of Vegetarian and Vegan Diets on the State of Bone Mineral Density in Humans. Crit. Rev. Food Sci. Nutr..

[25] Storz M.A., Ronco A.L. (2022). Carbohydrate Intake and Its Association with Dietary Acid Load in U.S. Adults: Results from a Cross-Sectional Study. Am. J. Lifestyle Med..

[26] Remer T., Manz F. (1994). Estimation of the Renal Net Acid Excretion by Adults Consuming Diets Containing Variable Amounts of Protein. Am. J. Clin. Nutr..

[27] Ekmeiro-Salvador J.E., Arévalo-Vera C.R. (2021). Vegetarianismo: Una caracterización antropométrica, dietética y motivacional en adultos venezolanos. RESPYN Rev. Salud Pública Nutr..

[28] Goodman D., González-Rivas J.P., Jaacks L.M., Duran M., Marulanda M.I., Ugel E., Mattei J., Chavarro J.E., Nieto-Martinez R. (2020). Dietary Intake and Cardiometabolic Risk Factors among Venezuelan Adults: A Nationally Representative Analysis. BMC Nutr..

[29] Food Processor—Nutrition Analysis Software for Dietitians|ESHA. ESHA Research. https://esha.com/products/food-processor/.

[30] Remer T., Manz F. (1995). Potential Renal Acid Load of Foods and Its Influence on Urine PH. J. Am. Diet. Assoc..

[31] Frassetto L.A., Todd K.M., Morris R.C., Sebastian A. (1998). Estimation of Net Endogenous Noncarbonic Acid Production in Humans from Diet Potassium and Protein Contents. Am. J. Clin. Nutr..

[32] Parmenter B.H., Dymock M., Banerjee T., Sebastian A., Slater G.J., Frassetto L.A. (2020). Performance of Predictive Equations and Biochemical Measures Quantifying Net Endogenous Acid Production and the Potential Renal Acid Load. Kidney Int. Rep..

[33] Parmenter B.H., Slater G.J., Frassetto L.A. (2017). Accuracy and Precision of Estimation Equations to Predict Net Endogenous Acid Excretion Using the Australian Food Database. Nutr. Diet..

[34] Esche J., Krupp D., Mensink G.B., Remer T. (2018). Dietary Potential Renal Acid Load Is Positively Associated with Serum Uric Acid and Odds of Hyperuricemia in the German Adult Population. J. Nutr..

[35] Farhangi M.A., Nikniaz L., Nikniaz Z. (2019). Higher Dietary Acid Load Potentially Increases Serum Triglyceride and Obesity Prevalence in Adults: An Updated Systematic Review and Meta-Analysis. PLoS ONE.

[36] Hejazi E., Emamat H., Sharafkhah M., Saidpour A., Poustchi H., Sepanlou S., Sotoudeh M., Dawsey S., Boffetta P., Abnet C.C. (2022). Dietary Acid Load and Mortality from All Causes, CVD and Cancer: Results from the Golestan Cohort Study. Br. J. Nutr..

[37] Joshi S., McMacken M., Kalantar-Zadeh K. (2021). Plant-Based Diets for Kidney Disease: A Guide for Clinicians. Am. J. Kidney Dis..

[38] Adair K.E., Bowden R.G. (2020). Ameliorating Chronic Kidney Disease Using a Whole Food Plant-Based Diet. Nutrients.

[39] Cosgrove K., Johnston C.S. (2017). Examining the Impact of Adherence to a Vegan Diet on Acid-Base Balance in Healthy Adults. Plant Foods Hum. Nutr..

[40] Knurick J.R., Johnston C.S., Wherry S.J., Aguayo I. (2015). Comparison of Correlates of Bone Mineral Density in Individuals Adhering to Lacto-Ovo, Vegan, or Omnivore Diets: A Cross-Sectional Investigation. Nutrients.

[41] Ströhle A., Waldmann A., Koschizke J., Leitzmann C., Hahn A. (2011). Diet-Dependent Net Endogenous Acid Load of Vegan Diets in Relation to Food Groups and Bone Health-Related Nutrients: Results from the German Vegan Study. Ann. Nutr. Metab..

[42] Müller A., Zimmermann-Klemd A.M., Lederer A.-K., Hannibal L., Kowarschik S., Huber R., Storz M.A. (2021). A Vegan Diet Is Associated with a Significant Reduction in Dietary Acid Load: Post Hoc Analysis of a Randomized Controlled Trial in Healthy Individuals. Int. J. Environ. Res. Public Health.

[43] Deriemaeker P., Aerenhouts D., Hebbelinck M., Clarys P. (2010). Nutrient Based Estimation of Acid-Base Balance in Vegetarians and Non-Vegetarians. Plant Foods Hum. Nutr..

[44] Xu H., Åkesson A., Orsini N., Håkansson N., Wolk A., Carrero J.J. (2016). Modest U-Shaped Association between Dietary Acid Load and Risk of All-Cause and Cardiovascular Mortality in Adults. J. Nutr..

[45] Penczynski K.J., Remer T., Menzel J., Abraham K., Weikert C. (2022). Urinary Potential Renal Acid Load (UPRAL) among Vegans Versus Omnivores and Its Association with Bone Health in the Cross-Sectional Risks and Benefits of a Vegan Diet Study. Nutrients.

