# Correction: Ekmeiro-Salvador, J.E.; Storz, M.A. The Impact of Plant-Based Diets on Dietary Acid Load Metrics in Venezuela: A Cross-Sectional Study. *Nutrients* 2023, *15*, 2745

**DOI:** 10.3390/nu17132077

**Published:** 2025-06-23

**Authors:** Jesús Enrique Ekmeiro-Salvador, Maximilian Andreas Storz

**Affiliations:** 1 Postgraduate Department, Food Science, University of Oriente, Anzoátegui 6001, Venezuela; jekmeiro@gmail.com; 2 Department of Internal Medicine II, Centre for Complementary Medicine, Freiburg University Hospital Faculty of Medicine, University of Freiburg, 79106 Freiburg, Germany

During a secondary data analysis toward the preparation of an additional manuscript, the authors identified an error in Figure 1 in the original publication [1], for which they contacted the Nutrients editorial office and requested the issuing of a corrigendum. The error was reported by the authors. The error does not affect the scientific content and conclusions of the manuscript. The error is discussed below. The authors of the manuscript sincerely apologize to the readers for these oversights.

## Error in Figure 1

In the original publication, there was a mistake in Figure 1 as published [1]. In this study, the authors analyzed nutrient intake data of *n* = 224 individuals. This was specified in Section 2.1, where the authors wrote, “In brief, we recruited *n* = 224 individuals that reported the consumption of a PBD between July 2018 and February 2020 in the metropolitan area of Puerto La Cruz in Venezuela, South America”. Regrettably, Figure 1 says that the final sample size was *n* = 221, instead. This is false and happened upon the creation of Figure 1. The correct sample size/sample number (*n* = 224) was correctly displayed in all tables and elsewhere in the manuscript. The corrected Figure 1 appears below.

## Error in Table 4

In the original publication, there was a mistake in Table 4 as published. In Table 4, model II for PRAL, a wrong standard error has been reported. The standard error has been reported as 1.42. In fact, this was wrong, and the correct standard error is 0.14. The corrected Table 4 appears below.

The authors state that the scientific conclusions are unaffected. This correction was approved by the Academic Editor. The original publication has also been updated.

## Figures and Tables

**Figure 1:**
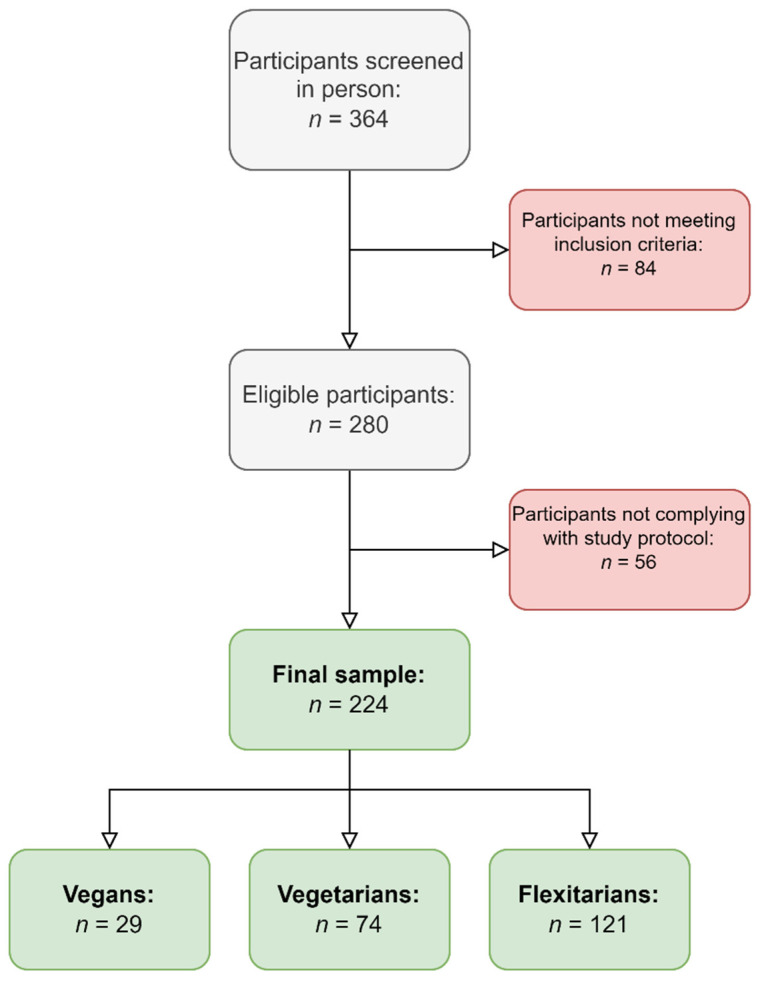
Participant inclusion flowchart.

**Table 4:** Multivariate linear regression models examining potential associations between PRAL_R_, NEAP_R_, NEAP_F_ (dependent variable, top, middle and bottom) and diet category.

Independent Variables	β	SE	*p*	β	SE	*p*
	**PRAL_R_**					
	Model I			Model II		
Diet Category						
Flexitarian	-	-	-	-	-	-
Lacto-Ovo-Vegetarian	−26.00	1.22	<0.001	−25.75	1.10	<0.001
Vegan	−44.42	1.71	<0.001	−42.82	1.60	<0.001
Sex						
Female				−3.06	1.04	0.003
Male				-	-	-
Body mass index				0.89	0.14	<0.001
	**NEAP_R_**					
	Model I			Model II		
Diet Category						
Flexitarian	-	-	-	-	-	-
Lacto-Ovo-Vegetarian	−26.24	1.59	<0.001	−25.85	1.13	<0.001
Vegan	−47.25	2.22	<0.001	−42.79	1.65	<0.001
Sex						
Female				−8.89	1.07	<0.001
Male				-	-	-
Body mass index				1.58	0.15	<0.001
	**NEAP_F_**					
	Model I			Model II		
Diet Category						
Flexitarian	-	-	-	-	-	-
Lacto-Ovo-Vegetarian	−15.68	0.44	<0.001	−15.58	0.38	<0.001
Vegan	−26.04	0.62	<0.001	−24.99	0.56	<0.001
Sex						
Female				−1.51	0.36	<0.001
Male				-	-	-
Body mass index				0.36	0.05	<0.001

Model I shows crude associations. Model II is adjusted for sex and BMI. Significant regression equations were found for PRAL_R_ (F(4,219) = 291.45, *p* < 0.001), with an R2 of 0.84; for NEAP_R_ (F(4,219) = 336.91, *p* < 0.001), with an R2 of 0.86; and for NEAP_F_ (F(4,219) = 821.79, *p* < 0.001) with an R2 of 0.94.

## References

[1] Ekmeiro-Salvador J.E., Storz M.A. (2023). The Impact of Plant-Based Diets on Dietary Acid Load Metrics in Venezuela: A Cross-Sectional Study. Nutrients.

